# High Prevalence of Gammaproteobacteria in the Sediments of Admiralty Bay and North Bransfield Basin, Northwestern Antarctic Peninsula

**DOI:** 10.3389/fmicb.2017.00153

**Published:** 2017-02-02

**Authors:** Diego C. Franco, Camila N. Signori, Rubens T. D. Duarte, Cristina R. Nakayama, Lúcia S. Campos, Vivian H. Pellizari

**Affiliations:** ^1^Departamento de Oceanografia Biológica, Instituto Oceanográfico, Universidade de São PauloSão Paulo, Brazil; ^2^Centro de Ciências Biológicas, Universidade Federal de Santa CatarinaFlorianópolis, Brazil; ^3^Departamento de Ciências Ambientais, Instituto de Ciências Ambientais, Químicas e Farmacêuticas, Universidade Federal de São PauloDiadema, Brazil; ^4^Departamento de Zoologia, Instituto de Biologia, Universidade Federal do Rio de JaneiroRio de Janeiro, Brazil

**Keywords:** marine sediments, microbial diversity, bacterial community structure, Antarctica, polar microbiology

## Abstract

Microorganisms dominate most Antarctic marine ecosystems, in terms of biomass and taxonomic diversity, and play crucial role in ecosystem functioning due to their high metabolic plasticity. Admiralty Bay is the largest bay on King George Island (South Shetland Islands, Antarctic Peninsula) and a combination of hydro-oceanographic characteristics (bathymetry, sea ice and glacier melting, seasonal entrance of water masses, turbidity, vertical fluxes) create conditions favoring organic carbon deposition on the seafloor and microbial activities. We sampled surface sediments from 15 sites across Admiralty Bay (100–502 m total depth) and the adjacent North Bransfield Basin (693–1147 m), and used the amplicon 454-sequencing of 16S rRNA gene tags to compare the bacterial composition, diversity, and microbial community structure across environmental parameters (sediment grain size, pigments and organic nutrients) between the two areas. Marine sediments had a high abundance of heterotrophic Gammaproteobacteria (92.4% and 83.8% inside and outside the bay, respectively), followed by Alphaproteobacteria (2.5 and 5.5%), Firmicutes (1.5 and 1.6%), Bacteroidetes (1.1 and 1.7%), Deltaproteobacteria (0.8 and 2.5%) and Actinobacteria (0.7 and 1.3%). Differences in alpha-diversity and bacterial community structure were found between the two areas, reflecting the physical and chemical differences in the sediments, and the organic matter input.

## Introduction

Marine microbial communities in sediments play a critical role in ecosystems functioning and are the main drivers of biogeochemical cycling of carbon, nitrogen and sulfur. They constitute a huge biomass portion of the Earth, are highly taxonomic diverse, and responsible for the majority of metabolic activity in the ocean including the polar regions (Parkes et al., 2000; Azam and Malfatti, 2007; Fuhrman, 2012). Various physical and chemical parameters can affect the marine sediment communities, in particular the organic matter. Studies conducted in the sediments of the Ross Sea (Carr et al., 2013) and the Drake Passage at the Antarctic Polar Front (Ruff et al., 2014) showed that the organic matter can increase the microbial abundance based on phospholipids and DNA sequencing, respectively. The organic matter can also influence communities composed by lithotrophic and heterotrophic microbes, associated with degradation of organics from sinking particles and fecal pellets in the Western Antarctica (Learman et al., 2016).

As consequences of climate change and rapid warming registered in the Western Antarctic Peninsula, the sea ice declines and glacial inputs increase. These conditions can favor phytoplankton and seaweed blooms (Meredith and King, 2005; Clarke et al., 2007; Montes-Hugo et al., 2009; Pearson et al., 2015), which then increase the quantity of organic matter transported from surface layers to the ocean floor (ex. Ducklow et al., 2006; Gillies et al., 2012). The quantity and type of carbon could also be altered due to the introduction of more terrestrial carbon into marine and benthic environments in polar systems (Learman et al., 2016).

Admiralty Bay is the largest bay on King George Island (South Shetlands Archipelago), located within the maritime Antarctic region. It was designated an Antarctic Specially Managed Area no. 1 (ASMA 1) and contains the Antarctic Specially Protected Area 128 (ASPA 128, former SSSI N° 8). Its hydrology is complex, as it receives different contributions from water masses originating in the Bransfield Strait, and also from ice melt within the bay (Szafrański and Lipski, 1982). Depending on the bathymetry, regional water circulation, winds and seasonal regime, waters from the Bransfield Strait that penetrate the bay originate from the adjacent warm and low saline Bellingshausen Sea (normally in the summer) or the cold and saline waters of Weddell Sea (in the winter) (Gordon and Nowlin, 1978; Tokarczyk, 1987; Huneke et al., 2016). The fjord-like shape of Admiralty Bay reflects freshwater input due to a strong glacial influence and high water column turbidity caused by suspension of soft sediments (Pichlmaier et al., 2004). All this organic matter falls to the seafloor, providing organic carbon to the reservoir, which is partially consumed by the cold-adapted microorganisms from the sediments, leading to low oxygen conditions (Orcutt et al., 2011).

To understand how the contribution of the organic matter from different sources can shape the microbial community in the Admiralty Bay and surrounding areas, in this study the bacterial diversity and community structure of the sediment samples from Admiralty Bay (AB) and adjacent areas of North Bransfield Basin (NBB) were compared. The results contribute to a better understanding of the sequenced microbial community structure in this polar area, and show differences in richness and community structure between the two sites across physical-chemical characteristics of the sediments.

## Materials and Methods

### Sampling Strategy

**FIGURE 1:**
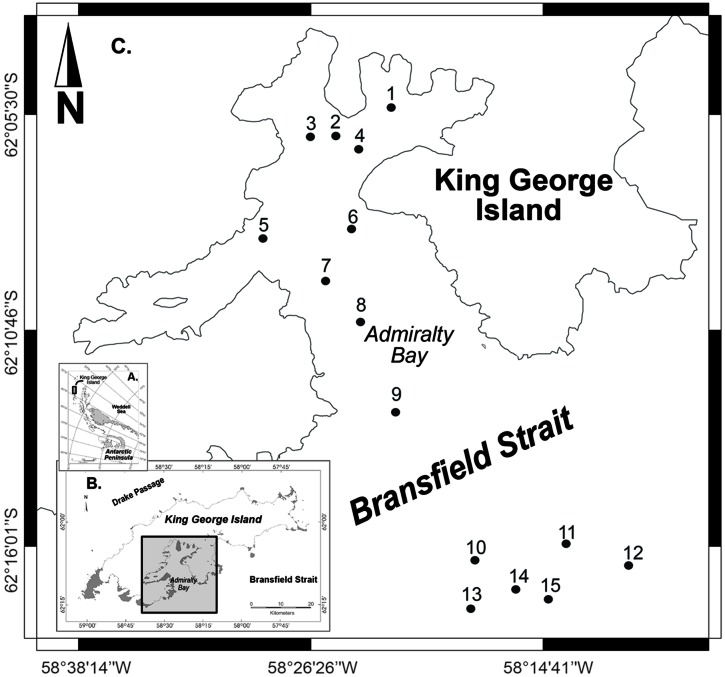
**Sampling map with 15 selected stations, 1–9 inside Admiralty Bay and 10–15 in the North Bransfield Basin, Northwestern Antarctic Peninsula**.

Surface sediment samples were collected along a bathymetric gradient in AB – King George Island (stations 1–9, depths ranging from 100 to 502 m) and NBB – Bransfield Strait (stations 10–15, depths 693–1,147 m), located in the Northwestern Antarctic Peninsula (**Figure 1**). Sampling was conducted by the Brazilian Navy vessel NApOc Ary Rongel during the austral summer, December 2008. In total, 15 samples of marine sediments were collected using a Mini Box Corer (MBC) at different depths. The top 5 cm of sediments were transferred to sterile Whirl-Pack sample bags (Nasco, WI, USA) and stored frozen onboard (-20°C). Samples were shipped to the University of São Paulo (USP) after 4 months of sampling.

### Environmental Parameters

Grain size was determined by laser diffraction (SALD 3101, Shimadzu, Japan), following the Wentworth scale (Suguio, 1973), yielding the mean and standard deviation of particle size (Folk and Ward, 1957), and classified into categories (clay, sand and silt). Concentrations of chlorophyll and phaeopigments were estimated according to Lorenzen (1967) and Plante-Cuny (1978), adapted for sediments (Gheller, 2014). Percentages of total organic carbon (TOC), total nitrogen, organic matter and carbonates were obtained using the CHNSO elemental analyzer (Elemental Combustion System 4010, Costech Analytical Technologies, USA).

### DNA Extraction, 16S rRNA Gene Amplification and Sequencing

Genomic DNA was extracted from 0.25 g of surface sediment in quadruplicate using a PowerSoil DNA Kit (MoBio, Carlsbad, CA, USA), according to the manufacturer’s instructions. Microbial 16S rRNA gene fragments were amplified using a set of primers designed by adding a 10-nucleotide barcode to the forward primer, 519F, (5′-CAGCMGCCGCGGTAATWC-3′) and reverse primer 1068R (5′-CTGACGRCRGCCATGC-3′) (Wang and Qian, 2009). The amplification reaction was carried out using the Accuprime *pfx* SuperMix (Thermo Scientific, USA) according to the manufacturer. PCR was performed with a thermal cycler (Thermo Scientific, USA) under the following conditions: 95°C for 5 min, 26 cycles of 95°C for 15 s, 59°C for 30 s and 68°C for 1 min. The PCR products were purified by using a DNA clean & concentrator kit (Zymo Research, USA). The amplicons from each sample were mixed at equimolar concentrations and then sequenced using GSFLX titanium instruments and reagents (Roche 454, Life Sciences, USA) at the Center for Advanced Technologies in Genomics (University of São Paulo, Brazil). All sequence data have been deposited in the National Center for Biotechnology Information Sequence Read Archives (SRA) under BioProject ID PRJNA335729.

### Sequencing Data Analyses

Raw sequence reads were filtered to eliminate the effect of the random sequencing using the Mothur 454 SOP (Schloss et al., 2011). The primer and barcodes of each read were removed and trimmed. Sequences shorter than 150 nucleotides with ambiguous bases or homopolymer regions were excluded. Sequences were clustered into operational taxonomic units (OTUs) by setting a 97% similarity. OTUs occurring once (singletons) were removed from dataset. Quality-filtered sequences were classified using the RDP Naïve Bayesian Classifier (Wang et al., 2007). For each sample, alpha-diversity indexes (Simpson diversity index and abundance-based coverage estimators - ACE index) were calculated using Mothur (version 1.35.1), and differences in alpha-diversity estimates between groups of samples were tested using Student *t*-test in R (version 3.3.2). The amplicon reads were normalized using the package DESeq2 (Love et al., 2014) following the general procedure for normalization using a variance stabilization transformation. DESeq2 normalized reads were used for all downstream analyses. The number of shared OTUs between samples were visualized using ggplot2 package in R (Wickham, 2009). Beta-diversity between samples was examined using Bray-Curtis dissimilarity matrix and ordinated by non-metric multidimensional scaling (nMDS) in R, with fitting of the environmental parameters applying the envfit function from the vegan package (Oksanen et al., 2013). To test the significance of differences between groups of samples (AB vs. NBB), analysis of similarity (adonis) was used.

## Results

### Environmental Parameters

Sediments in the area of study were mainly composed of silt and clay (representing 70.4 to 100.0% of sediment composition). In 12 of 15 samples, granulometry was dominated by silt, with the remaining three dominated by clay. The silt fraction in sediments varied from 39.1 to 64.9%, followed by clay (23.1–59.3%) and sand (0–29.5%) (**Table 1**). Stations located in the NBB had higher sand contents (6.6–20.6%) when compared to AB samples (0–10.1%), with the exception of station 5 (29.5%), located in front of Lange glacier.

**Table 1:** Environmental parameters measured for each sample in the sediments of Admiralty Bay and North Bransfield Basin, Western Antarctic Peninsula. Granulometry: Sand (%), Silt (%), Clay (%), Silt + Clay (%).

Sample	Sand(%)	Silt(%)	Clay(%)	Silt + Clay(%)	Chlorophyll(mg.m^-2^)	Phaeopigment (mg.m^-2^)	Chloro/PhaeoRatio	CPE	OM (%)	Carbonate (%)	TOC (%)	Total N (%)	C/NRatio
1	0.00	48.93	51.07	100.00	1.25	41.68	0.03	42.93	3.09	17.46	0.56	0.07	7.15


2	1.13	53.13	45.74	98.87	4.13	45.09	0.11	49.22	2.41	16.61	0.25	0.07	3.62


3	0.10	43.52	56.38	99.90	0.34	84.53	0.00	84.87	5.10	19.31	0.83	0.12	6.46


4	9.28	57.38	33.35	90.72	2.54	55.80	0.05	58.34	2.91	14.93	0.30	0.08	4.10


5	29.57	45.35	25.09	70.43	3.82	51.66	0.08	55.48	2.72	14.38	0.40	0.05	7.96


6	1.64	57.06	41.30	98.36	3.95	58.52	0.08	62.47	3.94	14.47	0.25	0.06	4.36


7	2.27	51.52	46.21	97.73	5.83	52.50	0.11	58.32	2.90	14.33	0.21	0.06	3.40


8	2.74	50.80	46.46	97.26	0.17	60.17	0.00	60.34	3.65	17.52	0.43	0.08	5.53


9	1.54	39.11	59.35	98.46	8.25	42.92	0.20	51.17	2.70	12.23	0.35	0.06	6.27


10	10.17	58.23	31.60	89.83	4.58	26.57	0.17	31.15	2.85	13.74	0.51	0.06	8.96
11	20.62	56.20	23.17	79.38	1.90	48.01	0.04	49.91	2.61	13.19	0.40	0.06	6.64
12	6.61	64.94	28.45	93.39	0.37	49.22	0.01	49.59	3.49	16.73	0.44	0.07	6.31


13	16.16	55.50	28.35	83.84	0.31	64.41	0.01	64.72	6.03	16.51	0.70	0.08	8.69


14	17.79	56.85	25.36	82.21	0.00	46.51	0.00	46.51	3.53	15.30	0.63	0.08	7.98


15	20.30	54.48	25.22	79.70	0.00	44.45	0.00	44.45	3.37	16.21	0.62	0.07	8.69

*Pigments: Chlorophyll (mg.m^-*2*^), Phaeopigment (mg.m^-*2*^), Chloro/Phaeo, Chlorophyll/Phaeopigment Ratio; CPE, Chloroplast Pigment Equivalent. OM, Organic Matter (%); Carbonate (%), TOC, Total Organic Carbon (%); Total N, Total Nitrogen (%); C/N, Total Carbon to Nitrogen Ratio*.

The sediment chlorophyll concentrations varied from zero (stations 14 and 15) to 8.25 mg.m^-2^ (station 9), and phaeopigment concentrations from 41.68 (station 1) to 84.53 mg.m^-2^ (station 3), indicating a predominance of degraded organic matter and small quantities of recent organic matter in the sediments. Stations in AB showed higher concentrations of chlorophyll than in the NBB, indicating the presence of higher quantities of recent organic matter to be degraded in the bay. Station 13 presented the highest organic matter concentration (6.0%) and C/N ratio (8.6).

### Community Composition

In this study, we used massively parallel signature sequencing technologies to obtain a total of 117,267 sequences (range 1000–26178 reads per sample) from 15 sediment samples from depths varying from 100 to 1.147 m. At the phylum level, all OTUs could be classified and belonged to 22 formally described bacterial phyla and 18 candidate phyla.

**FIGURE 2:**
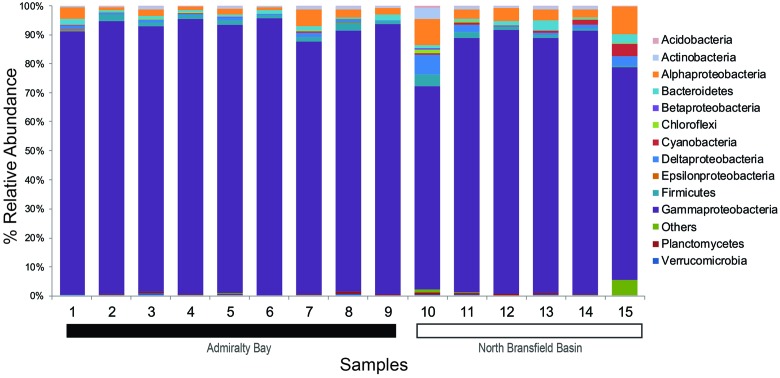
**Taxonomic composition and relative abundance of 16S rRNA sequences based on bacterial phyla and proteobacterial classes**. Samples 1–9 belong to Admiralty Bay, and 10–15 are from North Bransfield Basin.

The abundance analysis showed that eight phyla accounted for more than 97% of the total amplicons: Proteobacteria (89%), Firmicutes (1.5%), Bacteroidetes (1.4%), Actinobacteria (0.9%), Chloroflexi (0.7%), Planctomycetes (0.4%), Verrucomicrobia (0.3%) and Acidobacteria (0.1%) (**Figure 2**).

Considering the total of 40 classes identified, only 10 were found in all samples and accounted for 99.4% of the total tags. Based on the relative abundance, Gammaproteobacteria was the top dominant class in both sites, varying from 87.1 to 95.7% in AB and 70.5 to 91.4% in NBB. Alphaproteobacteria was the second dominant class, accounting for 0.9–5.9% in AB and 2.4–9.6% in NBB. Firmicutes (0.4–3.0% in AB and 0.4–4.0% in NBB) and Bacteroidetes (0.3–2.2% in AB and 0.6–3.4% in NBB) were also present in all sediment samples at similar rages. Other groups were more abundant in NBB in comparison with AB, such as Deltaproteobacteria (2.5 × 0.7%), Actinobacteria (1.3 × 0.7%) and Cyanobacteria (1.4 × 0.2%).

At the genus level, *Psychrobacter* showed high abundance in all 15 samples, varying from 79.3% (station 15) to 95.4% (station 4). Other nine abundant genera were *Psychromonas* (0–6.1%), *Gillisia* (0–4.1%), *Loktanella* (0–3.9%), *Paenisporosarcina* (0–2.4%), *Bacillariophyta* (0–1.8%), *Carnobacterium* (0–1.4%), *Planococcus* (0–1.1%), *Filomicrobium* (0–0.8%) and *Blastopirellula* (0–0.7%).

### Alpha Diversity

In general, alpha diversity values were higher in NBB when compared to AB. The number of observed OTUs per sample ranged from 36 (station 1) to 417 (station 10) (**Table 2**). The ACE index used for richness varied from 119.10 (station 1) to 1794.19 (station 11). Simpson’s diversity index varied from 0.40 (station 15) to 0.84 (station 9). Stations 11, 12, 15 and 10 (descending order), located in the NBB, showed the highest richness, and stations 15 and 10 presented the highest diversity. For ACE index, it was verified a significant difference between the two sites (*t*-test = 2.95, *p* = 0.02).

**Table 2:** Values of alpha-diversity estimates, using number of OTUs, ACE, Abundance-based Coverage Estimator and Simpson Index.

Sample	Latitude (°S)	Longitude (°W)	Depth (m)	Reads	Number of OTUs	ACE	Simpson Index	Coverage
1	62° 5′ 11.800′′	58° 22′ 19.300′′	100	1000	36	119.10	0.65	0.97
2	62° 6′ 14.000′′	58° 24′ 38.000′′	158	4728	80	247.32	0.73	0.98
3	62° 5′ 56.000′′	58° 26′ 15.000′′	105	8718	204	726.23	0.58	0.98
4	62° 6′ 15.800′′	58° 23′ 49.700′′	277	5004	76	261.54	0.79	0.99
5	62° 8′ 27.800′′	58° 28′ 33.200′′	296	8508	169	684.96	0.82	0.98
6	62° 8′ 17.700′′	58° 24′ 8.100′′	305	7970	108	378.77	0.77	0.99
7	62° 9′ 30.945′′	58° 25′ 25.501′′	493	11776	245	1217.65	0.73	0.98
8	62° 10′ 26.000′′	58° 23′ 41.001′′	502	5600	166	855.39	0.72	0.96
9	62° 12′ 38.888′′	58° 21′ 54.300′′	483	1894	50	288.52	0.84	0.98
10	62° 16′ 21.300′′	58° 18′ 3.003′′	693	5599	417	1520.40	0.42	0.95
11	62° 16′ 28.900′′	58° 10′ 10.800′′	724	14419	378	1794.19	0.75	0.98
12	62° 15′ 59.900′′	58° 13′ 29.800′′	708	26178	332	1744.01	0.80	0.99
13	62° 17′ 11.800′′	58° 15′ 50.002′′	1054	4722	141	464.45	0.72	0.98
14	62° 17′ 33.360′′	58° 18′ 12.600′′	1147	6161	194	706.26	0.72	0.97
15	62° 17′ 15.400′′	58° 14′ 26.100′′	1140	4990	341	1613.40	0.40	0.94
***T*-TEST^a^**
Average Admiralty Bay					83.56	364.63	0.74	
Average North Bransfield Basin					154.83	614.59	0.63	
t-statistics					3.45	2.95	1.32	
*P*-value					2.31	0.02	0.24	

^a^*Results of Student *t*-test, testing whether samples means of Admiralty Bay vs. North Bransfield Basin are different. *P*-value < 0.05 is significant*.

### Microbial Community Structure and the Influence of Environmental Parameters

**FIGURE 3:**
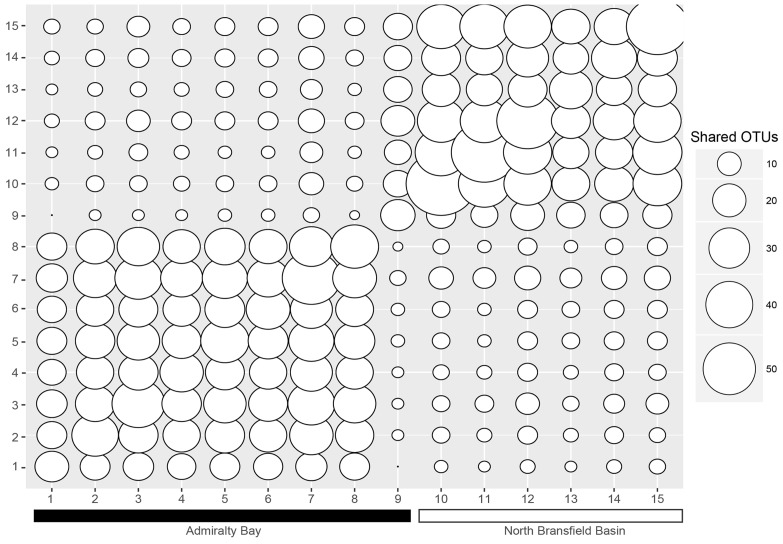
**Shared OTUs between the samples**. The numbers on the *x* and *y* axes correspond to the sample numbers (1–15). The smaller the circles, the less OTUs are shared, and the bigger the circles, the more OTUs are shared.

When analyzing the shared OTUs, it was found that more OTUs were shared within samples inside the bay *versus* samples outside the bay (**Figure 3**), except for station 9. Stations 2–8 shared more OTUs among each other, with sharing percentages varying from 48.3 to 92.8%. Station 9, placed at the entrance of the bay, shared 7–20 OTUs with stations 1–8, and 41–53 OTUs with stations 10–15, corresponding to 11.7–33.3% and 68.3–88.3%, respectively, and showing as a transitional environment. It was also evident that stations 10–15 shared more OTUs between each other (42.6–88.3%) than with stations inside the bay (3.8–33.3%).

To determine the distribution of the bacterial community (beta-diversity), the relative abundance of the different phyla and proteobacterial classes were analyzed in relation to the sampling locations and the sediment physical-chemical parameters using nMDS. It was revealed a clear distinction between samples collected inside AB and in the NBB, where stations 10 and 15 were more distant, which was statistically supported by adonis analysis (*r*^2^ = 0.20, *p* = 0.002). Only station 9, which is a transitional sampling point located at the entrance of the bay, was more related to the second group. Gammaproteobacteria was the dominant taxon for all samples, but others influenced station 10 (Firmicutes, Actinobacteria, Alphaproteobacteria, and Deltaproteobacteria) and station 15 (Deltaproteobacteria and others).

**FIGURE 4:**
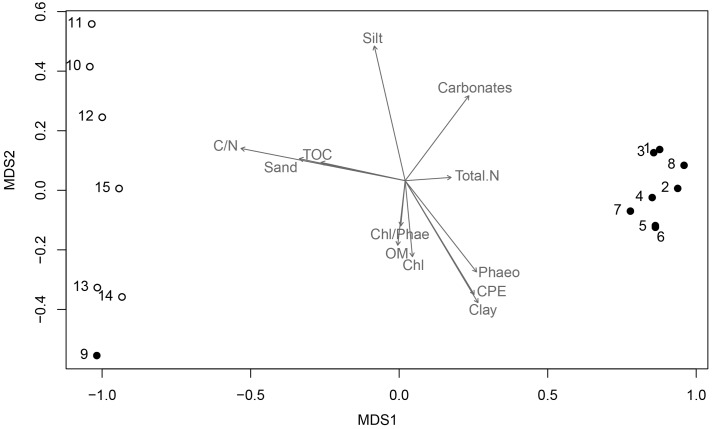
**Non-metric multidimensional scaling (nMDS) ordination**. Black dots represent stations 1–9 (Admiralty Bay), and white dots are stations 10–15 (North Bransfield Basin). Each arrow is significantly correlated to the ordination (envfit, *p* < 0.05), and represents the direction and strength of the environmental gradient (Clay, Silt, Sand, Phaeo, Phaeopigments; Chl, Chlorophyll; CPE, Chloroplast Pigment Equivalent, Carbonate; OM, Organic Matter; TOC, Total Organic Carbon; Total N, Total Nitrogen; C/N, Carbon to Nitrogen ratio).

The environmental factors associated with the sediment characteristics influenced the samples dissimilarity and the taxonomic composition. Sediments composed by clay, as well as relatively higher concentrations of phaeopigments were prevalent inside the bay and positively correlated with nMDS axis 1 (*r* = 0.62), whereas sediments constituted by sand and silt, and higher concentrations of total organic carbon and C/N ratio were predominant outside the bay (NBB), and negatively correlated with nMDS axis 1 (*r* = -0.98 for both parameters) (**Table 1**, **Figure 4**).

## Discussion

The present study compared the bacterial diversity and community structure found in marine sediments of AB and NBB. Although similar taxonomic groups were identified, mostly related to heterotrophic metabolism, their relative abundances, as well as alpha-diversity values and bacterial community structure have differed between the two sampling regions, possibly reflecting the distinct physical-chemical characteristics of the sediments (e.g., grain size, organic matter, chlorophyll, carbonate percentages, carbon and nitrogen concentrations). Additionally, hydro-oceanographic conditions such as bathymetry, regional water circulation and winds regime, continental inputs from glaciers melting, contribution of primary producers, seasonal entrance of water masses, and turbidity, might have favored the organic matter supply in the area. Indeed, a combination of physical and chemical parameters can influence the marine sediment community structure (e.g., Austen et al., 2002; Schauer et al., 2010; Zinger et al., 2011; Bienhold et al., 2012; Nguyen and Landfald, 2015; Learman et al., 2016).

The most abundant phylum (Proteobacteria) was represented by the classes Gamma-, Alpha- and Deltaproteobacteria in different proportions between the samples. This supports the results from previous studies showing that this phylum has a wide phylogenetic and phenotypic diversity in several marine and benthic environments (Sogin et al., 2006; Danovaro et al., 2010; Williams et al., 2010; Zinger et al., 2011; Lyra et al., 2013; Sun et al., 2013). Similarly, Proteobacteria has been reported from sediments of Antarctic Polar Front (Ruff et al., 2014), Ross Sea (Baldi et al., 2010) and Western Antarctic Peninsula (Learman et al., 2016).

The report of *Psychrobacter* as the dominant genus of Gammaproteobacteria in Antarctic sediments has not been previously reported. These heterotrophic versatile microorganisms include piezophilic and halophilic species (Kawasaki et al., 2002; Nogi et al., 2002), found in shallow and deep-sea sediments, in the water column, as part of the fish and krill microbiomes, and in association with brown macroalgae (Bozal et al., 2003; Lee et al., 2006; Dang et al., 2008; Teske et al., 2011; Tropeano et al., 2012). Most species of this genus can adapt to cold conditions, such as polar permafrost and ice, and are capable of reproducing at temperatures ranging from -10°C to 40°C (Rodrigues et al., 2009). They often produce low temperature-adaptive lipases (Bozal et al., 2003; Yumoto et al., 2003) and play essential roles in fat decomposition reactions (Li et al., 2013). The polar environment therefore constitutes an ecological niche for *Psychrobacter* strains (Bozal et al., 2003).

Alphaproteobacterial sequences affiliated with *Loktanella*, *Litorimicrobium* and *Hoefla*, which play a common role in the nitrogen cycle, were present in all samples, although in higher relative abundances outside the bay. They are able to reduce nitrate to nitrite, degrade aromatic compounds, and oxidize sulfur, ammonia, carbon monoxide, iron and manganese (Van Trappen et al., 2004a; Ivanova et al., 2005; Jin et al., 2011; Jung et al., 2013).

Flavobacteria, generally a major clade of Bacteroidetes in marine environments, was the third most abundant class. The clade was mainly represented by the genus *Gillisia*, specifically in samples 15 (4.1%) and 13 (3.4%), both located in NBB. Several strains have previously been isolated from marine and polar environments, such as Antarctic lakes (Van Trappen et al., 2004b). These heterotrophs are especially important, as they break down complex organic matter using exoenzymes to degrade algal cells and algal-detrital particles (Kirchman, 2002; Gómez-Pereira et al., 2012; Teeling et al., 2012). In fact, the presence of such distinct taxa in particular at stations placed outside the bay, indicated by alpha-diversity and community composition, helps to explain the differences in bacterial community between the two study areas.

The microbial community composition found in AB and NBB showed the prevalence of microorganisms related to heterotrophic metabolism. High relative abundance of heterotrophs can be explained by the high input of organic matter from different sources. The phytodetritus in deep sediments are allochthonous and can be derived from microphytobenthos, abundant in shallow areas of AB, or deposition of phytoplankton and macroalgae fragments (Gheller, 2014). Macroalgae can cover about 30% of the seabed surface of AB, and produce ca. 74,000 tons of wet biomass (Zielinski, 1990; Nedzarek and Rakusa-Suszczewski, 2004), resulting in substrates that favor the presence of these heterotrophs.

Phytoplankton blooms can also contribute to the transfer of organic matter to the seafloor. They normally occur in the study area during the austral summer due to the advection of continental input of ice melts (Nedzarek, 2008) and coastal upwelling (Brandini and Rebello, 1994; Schloss et al., 2002). This rapidly transported phytoplankton-derived organic carbon will be recycled by heterotrophic microbes fueling a diverse microbial community in deep sediments, as previously shown by Ruff et al. (2014). Moreover, the system of currents in the study area, influenced by the warmer and less saline waters from Bellingshausen Sea and the cold and saline waters from Weddell, may also indirectly influence microbial composition. The currents within the bay are more intense when compared to those in the inlets (Gordon and Nowlin, 1978; Huneke et al., 2016), thus enabling the transport, deposition and homogenization of the organic matter that drives the heterotrophic bacterial community.

## Conclusion

The next generation DNA sequencing data of 15 samples of seafloor sediments provides the first results characterizing the sediment microbial communities of the Northwestern Antarctic Peninsula, in a transect from AB to the NBB. Our study revealed high prevalence of heterotrophic gammaproteobacterial phylotypes, and differences in bacterial diversity and community structure between the two sites. A combination of conditions that favor the organic matter input, like regional water circulation and winds regime, bathymetry, continental influence from glaciers melting, inputs from primary producers of the euphotic zone or continental areas, water masses carrying nutrients, besides the grain size and sediment characteristics (organic matter, carbon and nitrogen concentrations), may contribute to shape the marine sediment communities in Antarctica.

## Author Contributions

DF, RD, and CS analyzed data and prepared figures and tables. All authors contributed to writing the paper.

## Conflict of Interest Statement

The authors declare that the research was conducted in the absence of any commercial or financial relationships that could be construed as a potential conflict of interest.
